# Autonomous replication sequences from the *Amaranthus palmeri* eccDNA replicon enable replication in yeast

**DOI:** 10.1186/s13104-020-05169-0

**Published:** 2020-07-10

**Authors:** William T. Molin, Allison Yaguchi, Mark Blenner, Christopher A. Saski

**Affiliations:** 1 Crop Production Systems Research Unit, USDA-ARS, Stoneville, MS 38776 USA; 2 Department of Chemical and Biomolecular Engineering, Clemson University, Clemson, SC 29634 USA; 3 Department of Plant and Environmental Sciences, Clemson University, Clemson, SC 29634 USA

**Keywords:** eccDNA replicon, Autonomous replication, EACS, DNA bending

## Abstract

**Objective:**

The objective of the research presented here was to determine whether autonomous replication sequences (ARS) discovered in the eccDNA replicon of glyphosate resistant *Amaranthus palmeri* enable self-replication in a yeast system.

**Results:**

Sequence analysis of the eccDNA replicon revealed a region of sharp changes in A + T/G + C content with characteristic bending indicative of an autonomous replication sequence. Further sequence analysis revealed an extended autonomous replication sequence (EACS) in close proximity to multiple DNA unwinding element (DUE) sequences. This region of the eccDNA replicon enabled autonomous replication of an ARS-less yeast plasmid.

## Introduction

Amplification of genes and gene clusters is a primary mechanism of genomic plasticity that is triggered in response to environmental stimuli. Recent reports have shown that these amplified genes and gene clusters are maintained as extra-chromosomal circular DNAs (eccDNAs) and are present across kingdoms. EccDNAs have been discovered in normal and cancerous human cells [1, 2], yeast [3], and plants [4–6]. In human cancers, eccDNAs have been shown to encode functional oncogenes, are present in multiple copies, and maintain highly accessible chromatin structures that promote long-range interactions with chromatin [7]. The presence of these eccDNAs across kingdoms suggests a fundamental biological role. In the plant species *Amaranthus palmeri* (S.) Wats., an eccDNA (the eccDNA replicon) was recently reported as the mechanism of amplification of the 5-enolpyruvylshikimate-3-phosphate synthase gene (*EPSPS*) that endows glyphosate resistance [4, 6]. Sequencing of the *EPSPS* eccDNA replicon revealed a large structure (~ 400 kb) with a complex repetitive architecture. The eccDNA replicon was reported to contain 58 genes in addition to the *EPSPS* gene, of which some had high constitutive expression profiles [6]. A genomic tethering mechanism was proposed that may use a protein intermediate, similar to human viruses, to mediate the association of the eccDNA replicon with nuclear chromatin as a means for genomic maintenance as part of the germline. Furthermore, a synteny/collinearity analysis with the chromosomal-scale reference assemblies of waterhemp (*A. tuberculatus*) and grain amaranth (*A. hypochondriacus*) revealed that the eccDNA replicon was likely the result of interactions among distal genomic regions, rather than a singular focal amplification surrounding the *EPSPS* gene. However, the mechanism by which the eccDNA replicon becomes amplified in copy number is not understood. Previous work has shown that genomic structure is associated with replication origins in plants [8], and that autonomously replicating sequences in plants have activity in yeast [9]. Here, we present an analysis of the eccDNA replicon that discovered sequences with a propensity for bending that are typically associated with autonomous replication sites and functional verification that these eccDNA sequences enable autonomous replication in yeast.

## Main text

### Methods

#### Sequence analysis of DNA A + T content and curvature

Genomic A + T and G + C content was globally determined for the eccDNA replicon by dividing the sequence into 50 bp sequential windows with the MakeWindows function of BedTools v.2.29.2 [10]. A + T and G + C content was determined for each window using the nuc function of BedTools v2.29.2 [10] and plotted as a circular track using the Circos v.0.69.9 [11]. DNA curvature analysis was performed by extracting a 256 bp segment from the eccDNA replicon sequence (coordinates: 287,484–287,739) and analyzed for curvature using the online version of dnacurve 2020.1.1 (17 Jan, 2020 release) [12] using the wedge model to determine the axial path of this segment of DNA [13]. The resulting protein data bank (PDB) file was visualized with the UCSF Chimera tool [14].

#### Cloning and functional verification of eccDNA autonomous replication sequences (ARS) sequences in yeast

The eccDNA ARS sequences were amplified using the 23A10 bacterial artificial chromosome (BAC) as template [15] using primers 167, 312F_CEN-SLIC and 168, 187R_SLIC (Additional file 1: Table S2). The yeast vector, pRS315, was linearized via PCR using primers pRS_ΔCEN-F and pRS_ΔARS-R such that the CEN6 sequence remained, but the native ARS was removed. Q5 polymerase was used for all PCRs. The eccDNA ARS was assembled into pRS305 and the ARS-less pRS315 using a sequence and ligation independent cloning (SLIC) reaction [16]. Constructs were confirmed with a restriction digest and sequencing. *Saccharomyces cerevisiae* (ATCC 208288) were transformed in triplicate as previously described [17]. Yeast cells were grown in a YPD (10 g/L yeast extract, 20 g/L peptone, 20 g/L glucose) preculture overnight at 28 °C and 250 rpm. In a 250-mL baffled flask, 50 mL of pre-warmed YPD was inoculated to a final titer of 5 × 10^6^ cells/mL. The culture was grown to a final titer of 2 × 10^7^ cells/mL at 28 °C and 250 rpm. Cells were harvested by centrifugation at 3000×*g* for 5 min. The cell pellet was resuspended in 25 mL of sterile milli-Q water and centrifuged again three times before cells were resuspended in 1.0 mL of sterile water. Cell pellet was then resuspended in 360 μL freshly made transformation mix (240 μL PEG 3350 (50% w/v), 34 μL 1.0 M LiAc, 50 μL single-stranded salmon sperm DNA (2 mg/mL), 36 μL plasmid DNA plus sterile water). Cells were heat shocked at 42 °C for 40 min and resuspended in 1 mL of sterile milli-Q water. 200 μL of cells were plated on YSC-Leu + 2% glucose plates and grown at 28 °C for 2 days and colonies counted. pRS305 lacks an ARS and served as a negative control while pRS315 contains an ARS and served as a positive control.

To confirm plasmid retention, colonies from transformed plates were passaged on YSC-Leu + 2% glucose plates three times. The passaged cells were used for a colony PCR using Q5 polymerase and primers ARS_cPCR-F and ARS_cPCR-R. The original colonies from the pRS305 transformation plate were used as templates, as they did not survive passaging. Positive controls using plasmids pRS305 + eccARS and pRS315 + eccARS and a negative control using wild type *S. cerevisiae* cells were performed. The PCR products were run on a 1% TAE gel with Genscript Ready-to-Use™ Plus 100 bp DNA Ladder.

### Results

#### Sequence analysis of DNA A + T content and curvature

**Fig. 1:**
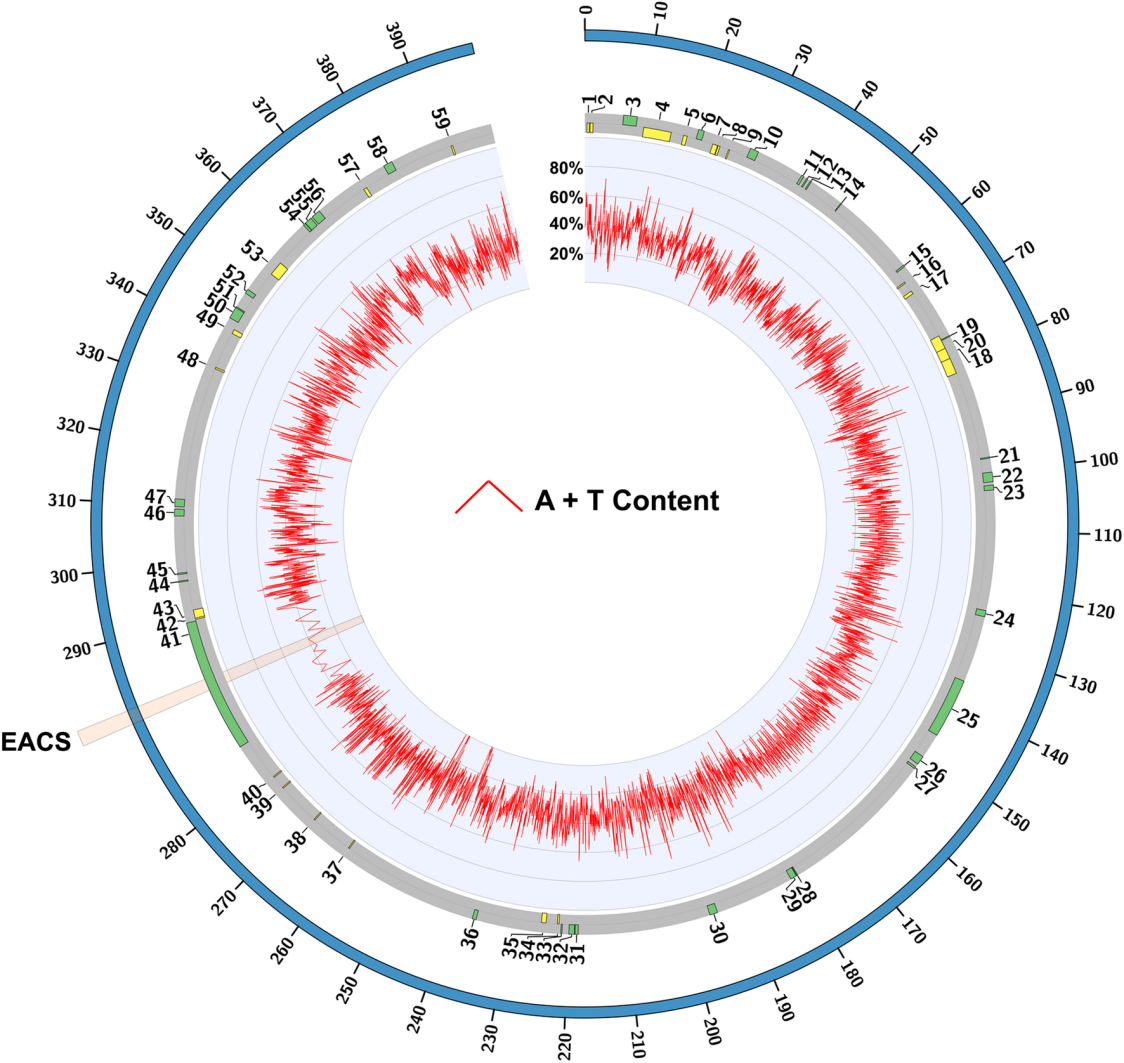
The eccDNA replicon reference sequence. Circular map of the eccDNA replicon with A + T content distribution determined as 50 bp sequential windows. The translucent highlight marks the single copy of the extended autonomous consensus sequence (EACS) in the NAC gene (*AP_R.00g000493*), number 41 on the map. This region on the circular map is zoomed in for high level detail of A + T content

**Fig. 2:**
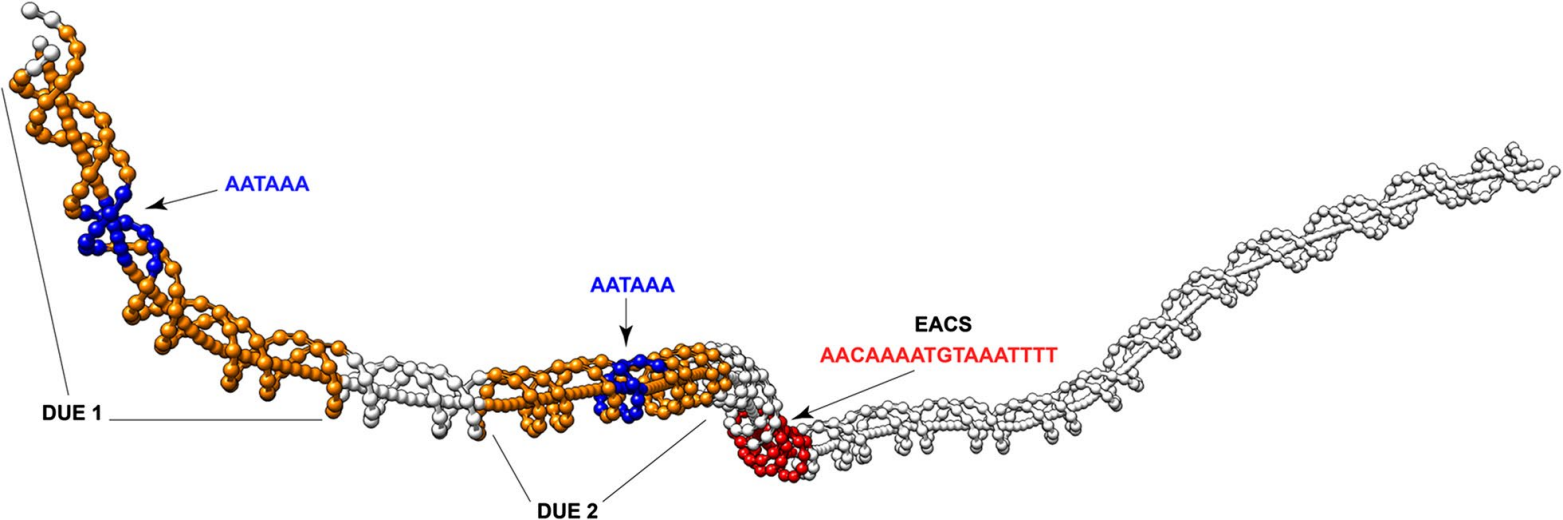
DNA bending profile of the EACS region. A 256 bp window of sequence of the EACS region of the eccDNA replicon. The extended autonomous consensus sequence (EACS) is highlighted in red and includes the 17 bp sequence with an exact match to the yeast ARS. DNA unwinding elements are highlighted in orange, with the conserved motif AATAAA in blue

**Fig. 3:**
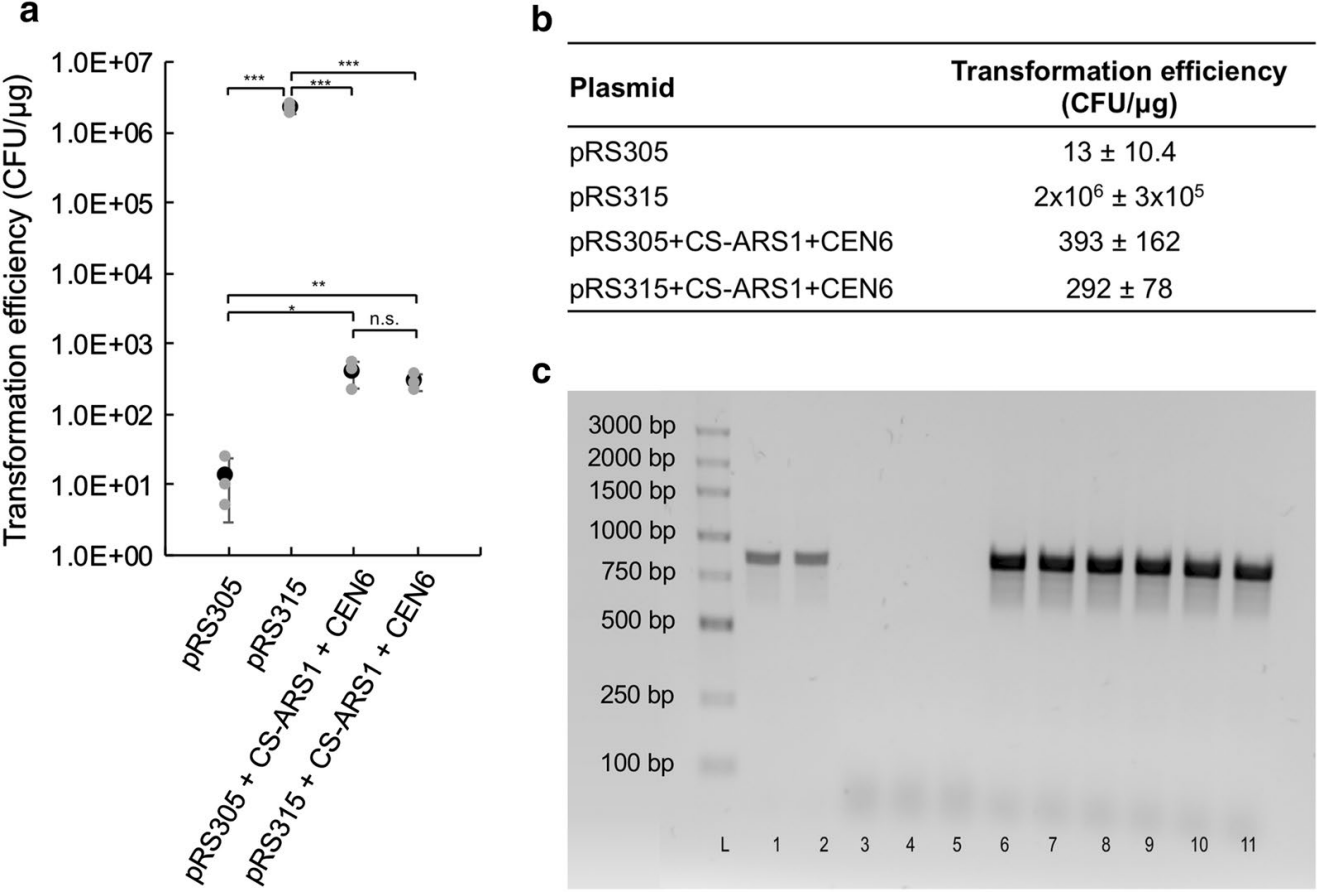
**a** Plotted transformation effiencies (CFU/μg) for pRS305, pRS315, pRS305 + CS-ARS1 + CEN6, and pRS315 + CS-ARS1 + CEN6. Statistical significance is noted by asterisks (***p-value = 0.0003; **p-value < 0.005; *p-value < 0.02; n.s., not significant). Smaller gray dots represent individual data points, larger black dot indicates sample average. Error bars represent standard deviation of the mean (n = 3 for all samples). **b** Tabulated data plotted in **a**. **c** Gel results for a colony PCR. Lanes are as follows: L, ladder; 1, pRS305 + CS-ARS1 + CEN6 plasmid; 2, pRS315 + CS-ARS1 + CEN6 plasmid; 3, wild type *S. cerevisiae* cells; 4, pRS305 transformed cells; 5, passaged pRS315 transformed cells, 6–8, triplicates of passaged pRS305 + CS-ARS1 + CEN6 transformed cells; 9–11, triplicates of passaged pRS315 + CS-ARS1 + CEN6 transformed cells

The eccDNA replicon is heavily punctuated with sharp changes in A + T and G + C content, which may imply biological function [18], including replication initiation sites [19] (Fig. 1). Overall, the eccDNA replicon biased in A + T content at 66%. A motif scan of the eccDNA replicon revealed a single exact match to the Extended Autonomous Consensus Sequence (EACS, 17 bp), previously described in yeast and other eukaryotes [20] (Fig. 1 and Additional file 2: Figure S1). A 50 bp window surrounding the EACS sequence was characteristically high in A + T content for an origin of replication at 76% (Additional file 2: Figure S1). Just upstream of the EACS sequence, two additional regions with elevated A + T content were also found and are characteristic of DNA unwinding elements (DUE) (Additional file 2: Figure S1). DUE 1 is 43 bp with 73% A + T content and DUE 2 is 41 bp in length and 62% A + T (Additional file 2: Figure S1). Nucleotide comparison of DUE 1 and DUE 2 did not reveal similar sequence, except for consistent elevated A + T content and a 6 bp AATAAA motif that is in common (Additional file 2: Figure S1). DNA curvature modeling of a 256 bp window containing the EACS and the two predicted DUE elements (287,484–287,739) revealed DNA bending, which is characteristic of reported origins of replication (Fig. 3 and Additional file 3: Table S1). There is consistent curvature from the beginning of DUE 1 to the end of DUE 2, with 2 sharper bends just in front of and after the EACS motif which is indicative of low helical stability (Fig. 2). Interestingly, this predicted origin of replication is contained within the eccDNA replicon gene, *AP_R.00g000493*, that contains a NAC domain. NACs are a large family of plant specific transcription factors whose functions include apical shoot development [21], secondary wall formation [22], and responses to abiotic/biotic pressures [23]. Interestingly, both the closely related genomes of waterhemp (*A. tuberculatus*), and grain amaranth (*A. hypochondriacus*) were searched and do not contain an annotated ortholog to the NAC gene. However orthologs were found in *Spinacia oleracea, Beta vulgaris*, and *Chenopodium quinoa*, which contained 2 copies. A nucleotide alignment of these sequences produced an overall pairwise identity of 63% with 756 identical sites, and an overall A + T content of 60% (Additional file 4: Figure S2). Both of the DUE sequences contain both insertions and deletions (indels) and single nucleotide variants (SNVs) in each of the three species, relative to the eccDNA replicon (Additional file 4: Figure S2). Interestingly, the EACS sequence was more conserved among the other species with seven variant positions across the 17 nucleotide consensus sequence. In addition, the other species sequences were less A + T rich when compared to the eccDNA replicon (Additional file 4: Figure S2).

#### Cloning and functional verification of EACS activity in yeast

By cloning ± 1 kb regions containing the putative origin of replication into a selectable ARS-less yeast vector, we observed dividing colonies, verifying that the eccDNA replicon ARS sequence is functional and can facilitate DNA replication in yeast (Fig. 3, Additional file 3: Table S1 and Additional file 5: Figure S3). Recombinant yeast growth was much slower with a lower abundance of colonies on plates with the eccDNA replicon ARS, relative to the control ARS suggesting a possible role of *cis*-elements and *trans*-factors for efficiency in the plant [19] (Additional file 5: Figure S3).

Transformation efficiencies for eccDNA ARS containing plasmids are approximately 300–400 CFU/μg, as compared to 2.15 × 10^6^ CFU/μg for pRS315 (Fig. 3). Transformation efficiencies between plasmids containing the eccDNA ARS are not statistically significant from each other, but are statistically different from the pRS305 and pRS315 plasmids, as determined by a two-tailed t-test with a 95% confidence level (Additional file 6: Figure S4). To validate plasmid retention and stability, cells were passaged on selective plates three times. Passaged cells were used in a colony PCR to validate retention of the ARS sequence (Fig. 3). Demonstration of bands in the cells transformed with plasmids containing CS-ARS1, and not in the wild type or cells transformed with pRS315, indicates dividing plasmids are due to the eccDNA ARS1.

### Discussion

The discovery of the eccDNA replicon as a mechanism of gene amplification is a remarkable and novel instance of genomic plasticity, however little is known about the mechanism of replication [4, 6]. Autonomous replicating sequences (ARS) can function as origins of replication in eukaryotes [20], and plants have been shown to have conserved ARS structures and sequence features commonly found in yeast and higher animals [8]. Additional reports have corroborated conserved ARS sequences in plants such as *Nicotiana tabacum* and Brassica species contain ARS sequences that function as a replication origin in yeast [9, 24, 25]. Characteristics of replication origins in plants include elevated A + T content coupled with DNA structure in the form of bending flanked by unusually straight structures [8]. In addition, sequence elements called DNA unwinding elements are common in both prokaryotes and eukaryotes. These regions are typically found in close proximity to ARS sequences, have elevated A + T content, and helical instability that can result in structure formation [26]. DUEs typically range in size from 30 to 100 bp without any typical consensus sequence [27, 28].

Here, we present an extension of our previous report that describes the sequence of the eccDNA replicon [6]. Sequence analysis of the eccDNA replicon found an exact match to the EACS sequence reported in yeasts and plants. A closer examination of this region identified two putative DNA unwinding elements in cis- organization to the EACS sequence with only a 6 bp nucleotide motif in common. These regions have high A + T content and display helical instability that can result in DNA structure. This DNA structure in the form of bending is thought to occur to facilitate accessibility of the DNA replisome [29], while the elevated A + T content is likely crucial for denaturation and maintenance of a stably unwound DNA structure during replication. DNA modeling also found two major bends that flank the EACS sequence coupled to straight DNA. This similar DNA structure being associated with autonomously replicating sequences from plants was reported in a study by Eckdahl et al. [8]. Cloning of the eccDNA replicon EACS + DUE segment produced dividing colonies in yeast when transformed into an ARS-less vector. Even though the eccDNA replicon contains an exact match to the yeast ARS sequence, the colony growth was much slower in comparison to the control (yeast native ARS) which suggests possible roles of cis/trans factors for efficiency in plants.

The origin of replication on the eccDNA replicon occurs in a putative NAC containing gene. The NAC containing gene family function in transcriptional regulation of plant stress response [23]. We identified genome-encoded NAC orthologs in distantly related species such as *Chenopodium*, *Spinacia*, and *Beta* but were unable to find an ortholog in the closely related *A. tuberculatus* and *A. hypochondriacus*. However, this could be an annotation related issue because a genomic region with evidence of similarity was found, but a gene was not predicted. Together these results advance our understanding of eccDNA function and replication origin in plants.

## Limitations

A well-defined consensus sequence has yet to be found for origins in any higher eukaryotic system, and yeast ARS sequences have never been shown to function in plant cells, to our knowledge. Further work is necessary to verify the function of these sequences in *A. palmeri*.

## Supplementary information

**Additional file 1: Table S2.** Yeast plasmids, strains, and primer sequences used to clone and validate the eccDNA ARS sequence.

**Additional file 2: Figure S1.** EACs sequence region of the eccDNA replicon. A zoomed in view of the extended autonomous consensus sequence (EACS) highlighted in red. Upstream, highlighted in orange, are the 2 predicted DNA unwinding elements (DUE), with the only conserved sequence within black bars (AATAAA).

**Additional file 3: Table S1.** DNA curvature results of a 256 bp window of sequence extracted from the eccDNA replicon that harbors 2 DNA unwinding elements (DUE) and the extended autonomous consensus sequence (EACS) with homology to yeast.

**Additional file 4: Figure S2.** NAC multiple sequence alignment. Multiple sequence alignment of the eccDNA replicon NAC gene that contains the EACS sequence. The EACS region is highlighted in red, and the DNA unwinding elements are highlighted in orange.

**Additional file 5: Figure S3.** Cloning of the eccDNA autonomous replication sequence in a yeast system. A. Water control (no colonies) B. pRS305 non-replicating plasmid (LEU marker replication − no colonies). C. pRS315 replicating plasmid (LEU marker, CEN6/ARS -lawn of colonies). D. pRS305 + CS-ARS1 + CEN6 (pRS305 + CEN6 + ARS1 − few colonies). E. pRS315 + CS-ARS1 + CEN6 (pRS315ΔARS + ARS1 − few colonies).

**Additional file 6: Figure S4.** Summary of the p-values resulting from two-tailed t-tests performed between samples using a 95% confidence level (α = 0.05).

## Data Availability

All data generated or analyzed during this study are included in this published article [and its additional information files].

## Abbreviations

eccDNA: Extrachromosomal circular DNA
EACS: Extended autonomous consensus sequence
DUE: DNA unwinding element
