# Supplementary figures and images for: Autonomous replication sequences from the Amaranthus palmeri eccDNA replicon enable replication in yeast
Source: BMC Res Notes. 2020 Jul 10;13:330. doi: 10.1186/s13104-020-05169-0 (PMC7350638; doi:10.1186/s13104-020-05169-0)

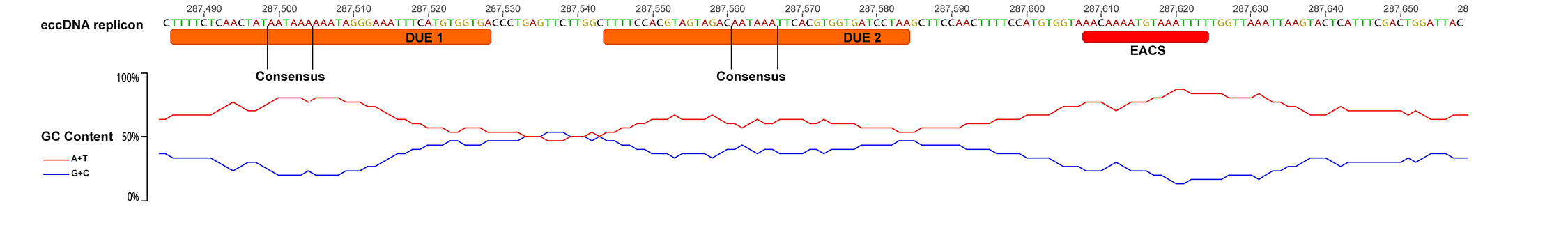

Supplement: Supplementary file 2 — Additional file 2: Figure S1. EACs sequence region of the eccDNA replicon. A zoomed in view of the extended autonomous consensus sequence (EACS) highlighted in red. Upstream, highlighted in orange, are the 2 predicted DNA unwinding elements (DUE), with the only conserved sequence within black bars (AATAAA). [file 13104_2020_5169_MOESM2_ESM.tif]

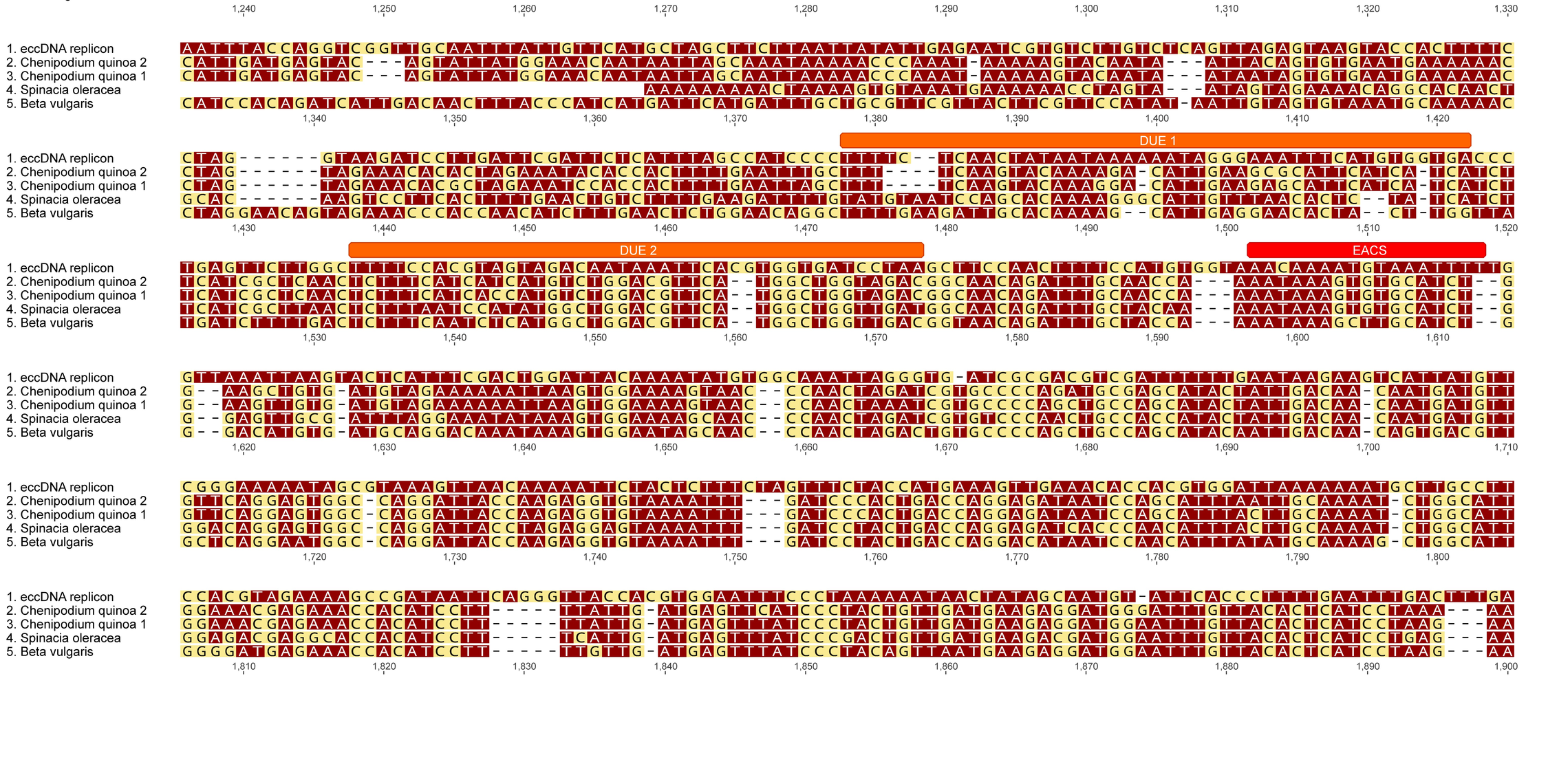

Supplement: Supplementary file 4 — Additional file 4: Figure S2. NAC multiple sequence alignment. Multiple sequence alignment of the eccDNA replicon NAC gene that contains the EACS sequence. The EACS region is highlighted in red, and the DNA unwinding elements are highlighted in orange. [file 13104_2020_5169_MOESM4_ESM.tif]

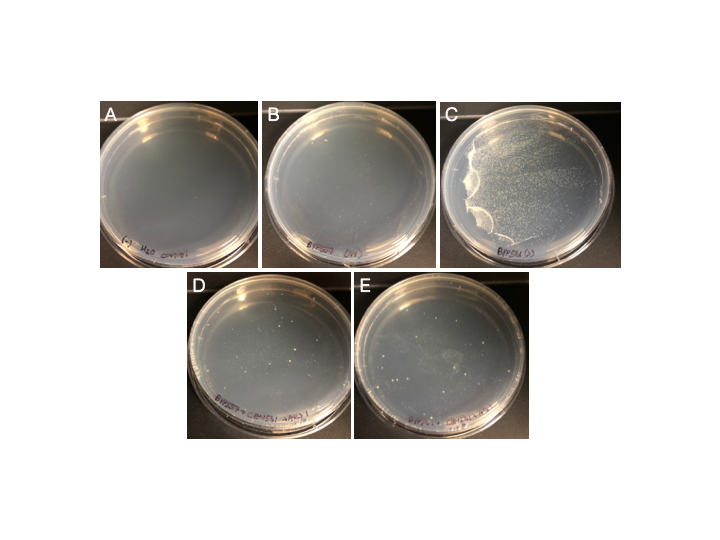

Supplement: Supplementary file 5 — Additional file 5: Figure S3. Cloning of the eccDNA autonomous replication sequence in a yeast system. A. Water control (no colonies) B. pRS305 non-replicating plasmid (LEU marker replication − no colonies). C. pRS315 replicating plasmid (LEU marker, CEN6/ARS -lawn of colonies). D. pRS305 + CS-ARS1 + CEN6 (pRS305 + CEN6 + ARS1 − few colonies). E. pRS315 + CS-ARS1 + CEN6 (pRS315ΔARS + ARS1 − few colonies). [file 13104_2020_5169_MOESM5_ESM.tif]

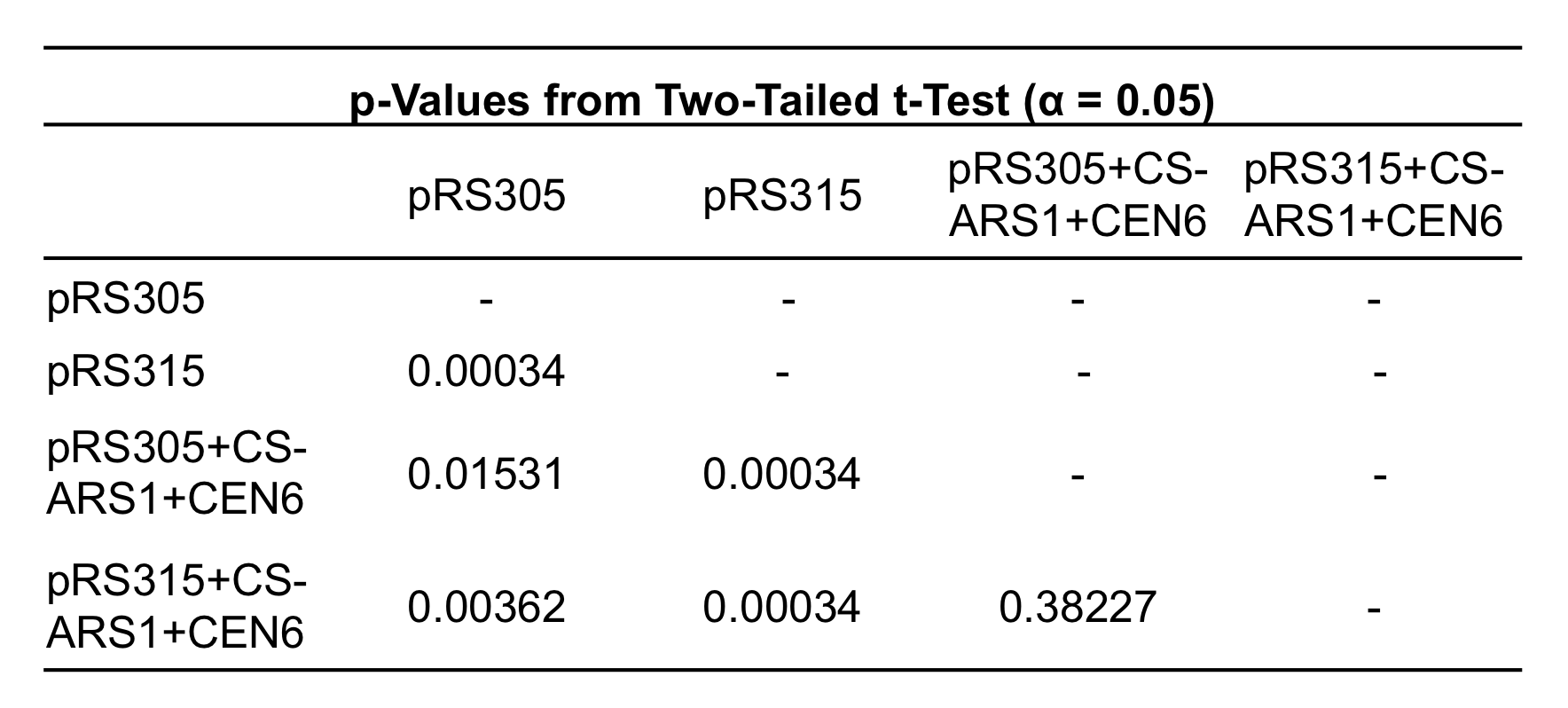

Supplement: Supplementary file 6 — Additional file 6: Figure S4. Summary of the p-values resulting from two-tailed t-tests performed between samples using a 95% confidence level (α = 0.05). [file 13104_2020_5169_MOESM6_ESM.tif]
